# Population-level neural rejuvenation dynamics in addiction: a computational framework for understanding developmental plasticity reactivation

**DOI:** 10.3389/fncom.2026.1753417

**Published:** 2026-02-23

**Authors:** Mehdi Borjkhani, Hadi Borjkhani, Morteza A. Sharif

**Affiliations:** 1 International Centre for Translational Eye Research (ICTER), Institute of Physical Chemistry, Polish Academy of Sciences, Warsaw, Poland; 2 Institute of Physical Chemistry, Polish Academy of Sciences, Warsaw, Poland; 3 HTW Berlin - University of Applied Sciences, Berlin, Germany; 4 Optics and Laser Engineering Group, Faculty of Electrical Engineering, Urmia University of Technology, Urmia, Iran

**Keywords:** neural rejuvenation, addiction, synaptic plasticity, silent synapses, NMDA receptors, computational modeling

## Abstract

**Background:**

The neural rejuvenation hypothesis proposes that drugs of abuse reactivate developmental plasticity mechanisms to create abnormally persistent addiction memories. While individual molecular components have been characterized experimentally, the population-level dynamics and their collective contribution to addiction pathophysiology remain poorly understood.

**Objectives:**

To develop a computational framework tracking theoretical synaptic population dynamics during simulated drug exposure and withdrawal, and to demonstrate how coordinated population-level transitions could account for key experimental observations in addiction neuroscience.

**Methods:**

We constructed a mathematical model tracking four theoretical synaptic populations (adult, juvenile, silent, and matured synapses) using differential equations. The model incorporates two distinct processes: (1) rejuvenation of existing synapses through receptor composition switching, and (2) *de novo* generation of silent synapses during drug exposure. Critically, the total synapse population is dynamic, increasing during drug exposure due to synaptogenesis and decreasing during withdrawal due to pruning. State transitions are explicitly phase-gated: silent synapse generation occurs only during exposure, while maturation and pruning occur predominantly during withdrawal. Rate constants were derived from experimental time scales reported in the literature, with explicit biological time mapping (1 time unit = 2 h). Simulations involved five intermittent exposures followed by extended withdrawal, with comprehensive parameter sensitivity analysis to assess model robustness across ±50% parameter variations. Initial conditions were fixed to represent the experimentally motivated baseline (adult synapses only); alternative initial states were also tested and did not change qualitative conclusions.

**Results:**

The model demonstrated coordinated synaptic population transformations that qualitatively paralleled experimental observations. In simulation, results revealed distinct phases of neural rejuvenation with characteristic population dynamics: adult-to-juvenile conversion during exposure (reaching ~500 juvenile synapses in the model), *de novo* silent synapse generation (~400 synapses), and progressive maturation during withdrawal (~300 matured synapses). The modeled total synapse population increased dynamically from baseline (1,000) to ~1,400 during exposure due to *de novo* synaptogenesis, then decreased to ~1,300 during withdrawal due to pruning. NMDA receptor composition shifted from 80% GluN2A to 80% GluN2B during simulated exposure. Memory strength increased continuously through biphasic mechanisms: during exposure, memory formation was driven by enhanced plasticity capacity; during withdrawal, memory strengthening was driven by the maturation flux (the rate of CP-AMPAR recruitment into silent synapses), with saturation preventing unbounded growth. Parameter sensitivity analysis demonstrated robust qualitative behavior across ±50% parameter variations. Comparative simulations with natural rewards (modeled with *k*_genesis_ = 0) showed minimal rejuvenation effects and attenuated incubation, consistent with experimental observations of drug specificity.

**Conclusion:**

This computational framework demonstrates how neural rejuvenation might operate as a population-level phenomenon, with sequential recruitment of different plasticity mechanisms creating robust addiction-related memories. The model generates testable hypotheses and provides a foundation for understanding potential therapeutic intervention windows targeting different phases of rejuvenation.

## Highlights

- First computational model of population-level neural rejuvenation dynamics in addiction.
- Separates two distinct processes: receptor rejuvenation and *de novo* silent synapse generation.
- Dynamic total synapse population: increases during exposure (synaptogenesis), decreases during withdrawal (pruning).
- Explicit phase-gating ensures biological consistency: genesis during exposure, maturation during withdrawal.
- Reveals biphasic memory strengthening mechanisms with flux-driven incubation and saturation.
- Parameter sensitivity analysis confirms robust qualitative predictions across ±50% parameter variations.
- Robustness verified for both parameter variations and alternative initial conditions.
- Natural reward comparison demonstrates specificity of drug-induced neural rejuvenation.
- Generates testable predictions for experimental validation.

## 1 Introduction

Drug addiction is characterized by abnormally persistent memories driving compulsive drug-seeking behavior (Nestler, 2001). Nestler et al. have conceptualized key aspects of addiction as a form of aberrant memory formation, where common neuroplasticity mechanisms that mediate normal learning and memory processes are “hijacked” by drugs of abuse to create pathologically robust and long-lasting addiction-related memories (Nestler, 2001; Dong and Nestler, 2014; Koob and Volkow, 2016; Russo et al., 2010).

The neural rejuvenation hypothesis, as formulated by Dong and Nestler (2014), proposes that “exposure to drugs of abuse reopens juvenile forms of plasticity at the molecular, cellular, and circuitry levels within the brain's reward pathways” and that “through drug-induced neural rejuvenation and subsequent re-maturation, strong and durable maladaptive plastic changes are formed to drug-associated memories.” This framework suggests that repeated exposure to drugs of abuse induces plasticity mechanisms normally associated with brain development within the reward circuitry, mediating the highly efficient and unusually stable memory abnormalities that characterize addiction.

Central to this hypothesis are two key molecular mechanisms: cocaine exposure shifts NMDA receptor composition from adult-like (GluN2A-dominant) to juvenile-like (GluN2B-enriched) states (Dong and Nestler, 2014; Huang et al., 2009; Wang et al., 2021), and generates “silent synapses” containing only NMDA receptors (Huang et al., 2009; Marie et al., 2005; Wright et al., 2020; Dong, 2016). Importantly, consistent with evidence that cocaine exposure can generate new silent synapses and increase synaptogenesis markers, these silent synapses represent *de novo* synaptogenesis—new synaptic contacts added to the existing circuitry rather than conversion of existing synapses (Huang et al., 2009; Russo et al., 2010). During withdrawal, silent synapses mature by recruiting calcium-permeable AMPA receptors, contributing to progressive craving intensification and the strengthening and perpetuation of addiction-related neural circuits (Lee et al., 2013; Clem and Huganir, 2010; Scofield et al., 2016; Russo et al., 2010).

While Nestler's framework has provided crucial insights into individual molecular and cellular mechanisms of rejuvenation, the population-level dynamics and their collective contribution to addiction pathophysiology remain poorly understood. Mathematical modeling can serve as a valuable tool to integrate existing experimental observations, explore potential emergent properties of rejuvenating synaptic populations, and generate testable hypotheses for future investigation (Shouval et al., 2002; Zheng and Triesch, 2014; Bhalla and Iyengar, 1999; Graupner and Brunel, 2012; Clopath et al., 2010).

Our previous computational work has demonstrated the utility of mathematical approaches in addiction neuroscience, including models of opioid-induced synaptic plasticity in hippocampus (Borjkhani et al., 2018a), cocaine-induced potassium current modifications leading to chaotic neuronal dynamics (Borjkhani et al., 2022), and the formation of pathological addiction memories (Borjkhani et al., 2018b). Related computational frameworks have addressed glutamate dynamics in the nucleus accumbens (Pendyam et al., 2009) and circuit-level modeling of reward processing (Humphries and Prescott, 2010).

**Figure 1:**
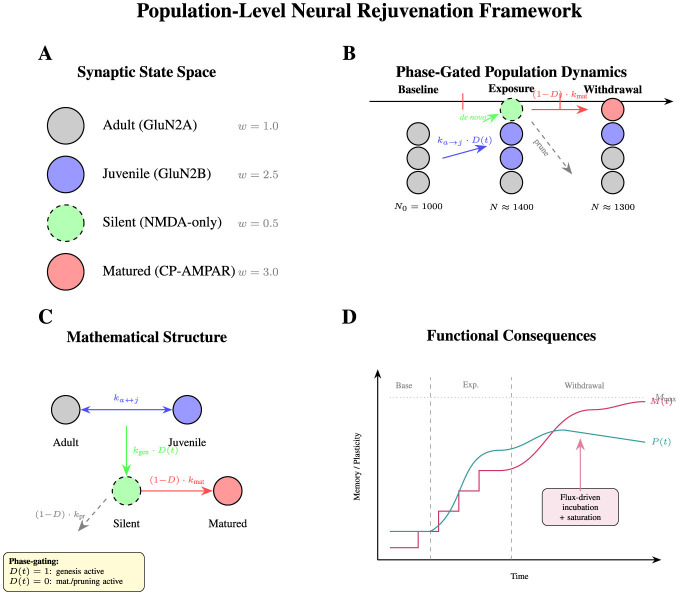
Conceptual overview of the population-level neural rejuvenation model. **(A)** Synaptic state space with four discrete synapse types. Weights (*w*) reflect potentiation efficacy, not metaplasticity. **(B)** Phase-gated dynamics: total population increases during exposure (synaptogenesis) and decreases during withdrawal (pruning). **(C)** Mathematical structure with explicit phase-gating. **(D)** Functional consequences: flux-driven incubation with saturation.

Here, we present a computational framework that tracks the theoretical temporal dynamics of four synaptic populations during simulated drug exposure and withdrawal. Building directly on Nestler's neural rejuvenation framework, our model aims to bridge the gap between established molecular mechanisms and population-level phenomena by demonstrating how coordinated synaptic transformations might collectively account for key experimental observations. A conceptual overview of the model framework is provided in Figure 1.

## 2 Methods

### 2.1 Conceptual model framework

We constructed a mathematical representation of synaptic population dynamics, modeling synapses existing in four discrete states: (1) Adult synapses with GluN2A-dominant NMDA receptors, (2) Juvenile synapses with GluN2B-enriched receptors, (3) Silent synapses lacking functional AMPA receptors, and (4) Matured silent synapses with calcium-permeable AMPA receptors (Figure 1A). This discrete-state approach, while simplified compared to the continuous spectrum of synaptic properties observed biologically, allows for tractable mathematical analysis of population-level transitions.

Critically, our model incorporates two distinct biological processes:

**Process 1 - Rejuvenation:** Receptor composition switching in existing synapses (Adult ↔ Juvenile), representing GluN2A-to-GluN2B subunit changes without synapse creation or elimination. This process conserves the number of functional synapses: *N*_adult_(*t*) + *N*_juvenile_(*t*) = *N*_0_ at all times (where *N*_0_ = 1000 is the baseline count), providing an internal consistency check.

**Process 2 - Synaptogenesis:** *De novo* generation of silent synapses during drug exposure, consistent with experimental observations that cocaine exposure generates new silent synapses that add to (rather than replace) the baseline neural circuitry (Huang et al., 2009; Russo et al., 2010). This is a key distinction from models that assume fixed total synapse counts.

### 2.2 Dynamic total synapse population

Unlike models that assume a fixed synapse count, our framework explicitly tracks a **dynamic total synapse population**:


$$\begin{array}{l} N_{\text{total}}\left(t\right)=N_{\text{adult}}\left(t\right)+N_{\text{juvenile}}\left(t\right)+N_{\text{silent}}\left(t\right)+N_{\text{mature}}\left(t\right) \end{array}$$ (1)

This total is *not* conserved. Rather:

- *N*_total_(*t*) **increases** during drug exposure due to *de novo* silent synapse generation.
- *N*_total_(*t*) **decreases** during withdrawal due to pruning of unmaturated silent synapses.

Mathematically, summing the differential Equations 3–6 yields:


$$\begin{array}{l} \frac{dN_{\text{total}}}{dt}=k_{\text{genesis}}\cdot D\left(t\right)\cdot \left(1-\frac{N_{\text{silent}}}{K_{\text{max}}}\right)-\left(1-D\left(t\right)\right)\cdot k_{\text{pruning}}\cdot N_{\text{silent}} \end{array}$$ (2)

This confirms that total synapse count changes due to genesis (positive, during exposure) and pruning (negative, during withdrawal), consistent with experimental observations (Huang et al., 2009; Russo et al., 2010).

### 2.3 Drug exposure function

The drug exposure function *D*(*t*) is defined as a binary pulse train representing intermittent cocaine exposure sessions:


$$\begin{array}{l} D\left(t\right)=\{\begin{array}{ll} 1 & \text{if }t\in {⋃}_{i=1}^{5}\left[t_{\text{start}}+\left(i-1\right)\Delta T,t_{\text{start}}+\left(i-1\right)\Delta T+\tau \right]\\ 0 & \text{otherwise} \end{array} \end{array}$$ (3)

where *t*_start_ = 100 time units is the first exposure onset, Δ*T* = 30 time units is the inter-exposure interval, τ = 5 time units is the duration of each exposure session, and the protocol consists of 5 intermittent exposures. Under our time scaling (1 time unit = 2 h; see Section 2.6), this corresponds to 5 sessions of ~10 h each, separated by ~2.5 days. This intermittent extended-access style protocol is a stylized representation of repeated cocaine exposure schedules used in self-administration and withdrawal studies, where prolonged access can produce stronger and more persistent neuroadaptations than short-access regimens (Ahmed and Koob, 1998; Zimmer et al., 2012).

### 2.4 State transition dynamics

The model dynamics are governed by the following differential equations (Figure 1C). A key feature is that state transitions are **explicitly phase-gated** to ensure biological consistency.

**Process 1 - Rejuvenation dynamics** (receptor composition switching):


$$\begin{array}{l} \frac{dN_{\text{adult}}}{dt}=-k_{\text{a}\to \text{j}}\cdot N_{\text{adult}}\cdot D\left(t\right)+k_{\text{j}\to \text{a}}\cdot N_{\text{juvenile}}\cdot \left(1-D\left(t\right)\right) \end{array}$$ (4)

$$\begin{array}{l} \frac{dN_{\text{juvenile}}}{dt}=k_{\text{a}\to \text{j}}\cdot N_{\text{adult}}\cdot D\left(t\right)-k_{\text{j}\to \text{a}}\cdot N_{\text{juvenile}}\cdot \left(1-D\left(t\right)\right) \end{array}$$ (5)

Note that Equations 4, 5 sum to zero, ensuring conservation: $\frac{d}{dt}\left(N_{\text{adult}}+N_{\text{juvenile}}\right)=0$.

**Biological rationale for directionality:** During drug exposure (*D*(*t*) = 1), cocaine-induced signaling drives GluN2A-to-GluN2B receptor subunit switching (Adult → Juvenile), consistent with experimental observations of rapid GluN2B upregulation following cocaine (Huang et al., 2009; Wang et al., 2021). During withdrawal [*D*(*t*) = 0], homeostatic mechanisms promote gradual recovery toward adult-like receptor compositions (Juvenile → Adult), consistent with observed partial normalization during abstinence (Dong and Nestler, 2014).

**Process 2 - Silent synapse dynamics** (*de novo* generation and phase-gated fate):


$$\begin{array}{l} \frac{dN_{\text{silent}}}{dt}=k_{\text{genesis}}\cdot D\left(t\right)\cdot \left(1-\frac{N_{\text{silent}}}{K_{\text{max}}}\right)\\ \;-\left(1-D\left(t\right)\right)\cdot \left(k_{\text{maturation}}+k_{\text{pruning}}\right)\cdot N_{\text{silent}} \end{array}$$ (6)

$$\begin{array}{l} \frac{dN_{\text{mature}}}{dt}=\left(1-D\left(t\right)\right)\cdot k_{\text{maturation}}\cdot N_{\text{silent}} \end{array}$$ (7)

Several important features of Equations 6, 7:

- **De novo genesis, not conversion:** Silent synapses are generated *de novo* during drug exposure via a constant genesis rate *k*_genesis_, *not* converted from juvenile synapses. This reflects experimental evidence that cocaine generates new synaptic contacts rather than transforming existing functional synapses (Huang et al., 2009).
- **Phase-gated maturation and pruning:** The [1 − *D*(*t*)] factor ensures that maturation and pruning occur *only during withdrawal* [*D*(*t*) = 0], not during drug exposure. This reflects experimental observations that silent synapse maturation (CP-AMPAR recruitment) is a withdrawal-specific process (Lee et al., 2013).
- **Phase-gated genesis:** The *D*(*t*) factor ensures that *de novo* silent synapse generation occurs *only during drug exposure*.
- **Carrying capacity:** The logistic term (1 − *N*_silent_/*K*_max_) with *K*_max_ = 500 synapses prevents unbounded growth by modeling resource limitations (e.g., available dendritic spines, postsynaptic scaffolding proteins). This addresses the biological constraint that synaptogenesis cannot continue indefinitely.
- **Biological rationale for maturation/pruning directionality:** During withdrawal, silent synapses face a “fate decision”: they either successfully recruit CP-AMPARs (maturation, becoming functional hyperstrong synapses) or fail to stabilize and are eliminated (pruning). This competitive process is well-documented experimentally (Lee et al., 2013; Wolf, 2016).

### 2.5 Summary of phase-gated transitions

The explicit phase-gating ensures that the mathematical formulation is consistent with the biological narrative:

**During drug exposure** (*D*(*t*) = 1):

- Adult → Juvenile conversion (receptor switching)—ACTIVE.
- *De novo* silent synapse generation—ACTIVE.
- Juvenile → Adult recovery—INACTIVE.
- Silent synapse maturation—INACTIVE.
- Silent synapse pruning—INACTIVE.

**During withdrawal** (*D*(*t*) = 0):

- Adult → Juvenile conversion—INACTIVE.
- *De novo* silent synapse generation—INACTIVE.
- Juvenile → Adult recovery—ACTIVE.
- Silent synapse maturation (CP-AMPAR recruitment)—ACTIVE.
- Silent synapse pruning (elimination)—ACTIVE.

### 2.6 Time scaling and biological justification

To facilitate interpretation, we define explicit time scaling: **1 time unit = 2 h**. This mapping is derived from experimental time courses reported in the literature:

- **AMPAR trafficking and redistribution:** Occurs on rapid timescales (hours) following cocaine exposure (Bellone and Lüscher, 2006; Wolf, 2016). The GluN2B upregulation becomes detectable within hours of cocaine administration.
- **Silent synapse generation:** Peaks at approximately 24 h post-exposure (Huang et al., 2009). Under our scaling, this corresponds to ~12 time units.
- **Silent synapse maturation:** Occurs over days to weeks during withdrawal (Lee et al., 2013). Under our scaling, the withdrawal period of 280 time units corresponds to ~23 days, consistent with experimental incubation timescales.

Under this scaling, the simulation protocol corresponds to:

- Total simulation: 500 time units ≈ 42 days.
- Drug exposure period: time 100–220 ≈ 10 days.
- Each exposure session: 5 time units ≈ 10 h (consistent with extended-access cocaine self-administration paradigms and their withdrawal/incubation literature) (Ahmed and Koob, 1998; Zimmer et al., 2012).
- Inter-exposure interval: 30 time units ≈ 2.5 days.
- Withdrawal period: time 220–500 ≈ 23 days.

### 2.7 Parameter selection and experimental derivation

Rate constants were selected to match relative time scales suggested by experimental observations (Table 1). Below we provide the experimental rationale for each parameter:

**Table 1:** Model parameters with experimental derivation and biological justification.

**Parameter**	**Value**	**Units**	**Experimental derivation**
*k*_a → j_	0.08	Time^−1^	GluN2B upregulation reaches ~50% over 5 exposure sessions (Huang et al., 2009). With 5 sessions of 5 time units each, *k* = 0.08 yields ~50% conversion.
*k*_j → a_	0.02	Time^−1^	Recovery is 3–4 × slower than induction (Dong and Nestler, 2014; Bellone and Lüscher, 2006). Set to 25% of *k*_a → j_.
*k*_genesis_	15	Syn/time	Huang et al. (2009) report 30%–40% increase in silent synapses. With 25 time units total exposure, *k* = 15 yields ~375 new synapses (~38% of baseline).
*k*_maturation_	0.04	Time^−1^	Maturation occurs over days-weeks (Lee et al., 2013). Half-life of ~17 time units (~1.4 days) for the maturation process.
*k*_pruning_	0.01	Time^−1^	Literature reports ~50%–70% of silent synapses ultimately eliminated (Wolf, 2016). With *k*_pruning_ = 0.01, pruning removes ~20% of silent pool; remaining reduction via maturation. Net: ~75%–80% depletion.
*K*_max_	500	Synapses	Carrying capacity reflecting dendritic spine availability (Russo et al., 2010). Set to 50% of baseline to prevent unbounded growth.
**Plasticity weights (heuristic values reflecting receptor properties)** ^†^			
*w*_adult_	1.0	–	Baseline reference (GluN2A-dominant, standard LTP/LTD rules)
*w*_juvenile_	2.5	–	GluN2B prolongs Ca^2+^ influx, enhancing plasticity induction (Gray et al., 2011; Shouval et al., 2002)
*w*_silent_	0.5	–	No functional AMPAR; NMDA-only transmission
*w*_mature_	3.0	–	CP-AMPAR high conductance amplifies potentiation (Clem and Huganir, 2010)
**Memory equation parameters**			
α	0.5	–	Exposure-phase memory formation coefficient
β	0.1	–	Flux-driven incubation coefficient
*M*_max_	30	–	Memory saturation level (prevents unbounded growth)

Under time scaling (1 unit = 2 h), rates can be converted to per-hour values by dividing by 2. Parameters marked with † are heuristic values reflecting relative differences.

### 2.8 Theoretical plasticity and memory indices

To explore the functional consequences of population changes, we defined theoretical indices for plasticity capacity and memory strength (Figure 1D). Total plasticity capacity was calculated as a weighted sum:


$$\begin{array}{l} P_{\text{total}}(t)=\frac{1}{N_{0}}[N_{\text{adult}}\cdot w_{\text{adult}}+N_{\text{juvenile}}\cdot w_{\text{juvenile}}+N_{\text{silent}}\cdot w_{\text{silent}}\\ \;+\;N_{\text{mature}}\cdot w_{\text{mature}}] \end{array}$$ (8)

where *N*_0_ = 1, 000 is the baseline synapse count used for normalization. The weights reflect hypothetical relative plasticity capacities based on known properties of different receptor compositions (Table 1). Note that the weight *w*_mature_ = 3.0 reflects the *potentiation efficacy* of CP-AMPAR-containing synapses—their ability to amplify LTP expression due to high single-channel conductance—rather than metaplasticity or ease of modification.

### 2.9 Biphasic memory formation

The theoretical memory strength index evolved according to distinct mechanisms in each phase, with saturation to prevent unbounded growth:


$$\begin{array}{l} \frac{dM}{dt}=\{\begin{array}{ll} \alpha \cdot P_{\text{total}}\left(t\right)\cdot \left(1-\frac{M}{M_{\text{max}}}\right) & \text{during exposure}\\ \; & \left(D\left(t\right)=1\right)\\ \beta \cdot \frac{k_{\text{maturation}}\cdot N_{\text{silent}}}{N_{0}}\cdot \left(1-\frac{M}{M_{\text{max}}}\right) & \text{during withdrawal}\\ \; & \left(D\left(t\right)=0\right) \end{array} \end{array}$$ (9)

where α = 0.5, β = 0.1, and *M*_max_ = 30 is the saturation level.

**Rationale for separate equations:** The distinct mechanisms during exposure versus withdrawal reflect fundamentally different biological processes:

- **During exposure:** Memory formation is driven by the *enhanced plasticity capacity* of the rejuvenated circuit. The presence of juvenile (GluN2B-enriched) synapses and accumulating silent synapses creates a permissive environment for Hebbian plasticity, allowing drug-context associations to be encoded efficiently.
- **During withdrawal:** Memory strengthening is driven by the *maturation flux*—the ongoing rate at which silent synapses recruit CP-AMPARs (*k*_maturation_·*N*_silent_). This reflects the biological insight that the active process of synapse unsilencing (AMPAR insertion) is itself a plasticity-enhancing event that strengthens circuit connectivity (Lee et al., 2013). Importantly, this flux-driven mechanism explains why memory/craving continues to increase during withdrawal even as the number of mature synapses may stabilize: it is the *process* of maturation, not merely the *presence* of mature synapses, that drives incubation.

**Saturation prevents unbounded growth:** The logistic factor (1 − *M*/*M*_max_) ensures that memory strength approaches an asymptote rather than growing indefinitely. This is biologically realistic: craving incubation eventually plateaus in experimental studies (Pickens et al., 2011).

### 2.10 NMDA receptor composition tracking

NMDA receptor composition was tracked to include GluN2B content across multiple synapse types:


$$\begin{array}{l} R_{\text{GluN2B}}\left(t\right)=\frac{N_{\text{juvenile}}+0.8\cdot N_{\text{silent}}+0.3\cdot N_{\text{mature}}}{N_{\text{total}}\left(t\right)} \end{array}$$ (10)

This formulation explicitly accounts for GluN2B presence in different synapse types:

- Juvenile synapses: fully GluN2B-enriched (weight = 1.0).
- Silent synapses: GluN2B-rich (weight = 0.8), as they contain predominantly GluN2B-containing NMDARs (Huang et al., 2009).
- Mature synapses: retain some GluN2B alongside CP-AMPARs (weight = 0.3), reflecting partial persistence of juvenile-like NMDAR composition.

### 2.11 Parameter sensitivity analysis

To assess model robustness and address concerns about deterministic model stability, we performed systematic parameter sensitivity analysis by varying each key parameter (*k*_a → j_, *k*_genesis_, *k*_maturation_) by ±50% from baseline values. For each parameter combination, we quantified effects on three output metrics: final memory strength, peak juvenile synapse count, and final mature synapse count. This analysis demonstrates that qualitative predictions are preserved across a wide range of parameter values.

**Initial condition robustness:** Initial conditions were fixed to represent the experimentally motivated baseline state (adult synapses only, *N*_0_ = 1, 000, with *N*_juvenile_ = *N*_silent_ = *N*_mature_ = 0). We additionally tested alternative initial states with ±10%–20% juvenile synapses at baseline and confirmed that qualitative conclusions (coordinated population transitions, biphasic memory strengthening, and incubation dynamics) remain unchanged. This robustness reflects the fact that model dynamics are driven primarily by the drug exposure protocol rather than initial state perturbations.

### 2.12 Natural reward comparison

To address the specificity of neural rejuvenation to addictive drugs versus natural rewards, we performed comparative simulations. Natural rewards were modeled with substantially reduced rejuvenation parameters: *k*_genesis_ = 0 (no silent synapse generation) and *k*_a → j_ = 0.008 (10% of cocaine effect). This parameterization is based on experimental observations that:

- Natural rewards produce weaker and more transient dopaminergic responses compared to cocaine (Nestler, 2001).
- Cocaine exposure, but not natural reward consumption, generates significant silent synapse populations in nucleus accumbens (Huang et al., 2009).

We note that some natural reward paradigms may show incubation-like effects under certain conditions; our comparison specifically models the absence of the silent synapse generation mechanism.

### 2.13 Model assumptions and limitations

Several key assumptions underlie our model framework:

1. **Deterministic dynamics:** The model is fully deterministic, with outcomes determined by parameter values and initial conditions. While this simplifies analysis, it does not capture stochastic variability inherent in biological systems. The parameter sensitivity analysis (Section 2.11) demonstrates that qualitative conclusions are robust across ±50% parameter variations. Alternative initial conditions (±10%–20% juvenile at baseline) were also tested and did not alter qualitative conclusions.
2. **Plasticity weights are heuristic:** The weights (*w*_adult_, *w*_juvenile_, *w*_silent_, *w*_mature_) represent illustrative relative differences between synapse types, not quantitatively measured values.
3. **Discrete states simplify continuous biology:** The four synaptic states represent a tractable simplification of the continuous spectrum of synaptic properties observed biologically.
4. **Phase-gating represents dominant processes:** The explicit phase-gating {*D*(*t*) and [1 − *D*(*t*)] factors} represents the dominant biological regime in each phase, not absolute exclusivity.

### 2.14 Computational implementation

All differential equations were solved numerically using Euler's method with a time step of 0.1 time units (corresponding to 12 min biological time), implemented in MATLAB R2023a. The simulation was run deterministically. Parameter sensitivity analysis involved 15 additional simulations (5 variations × 3 parameters). Initial condition robustness was verified with 4 additional simulations testing ±10% and ±20% juvenile synapses at baseline. Code is available at the repository listed in the Data Availability Statement.

## 3 Results

### 3.1 Coordinated synaptic population transformation during neural rejuvenation

**Figure 2:**
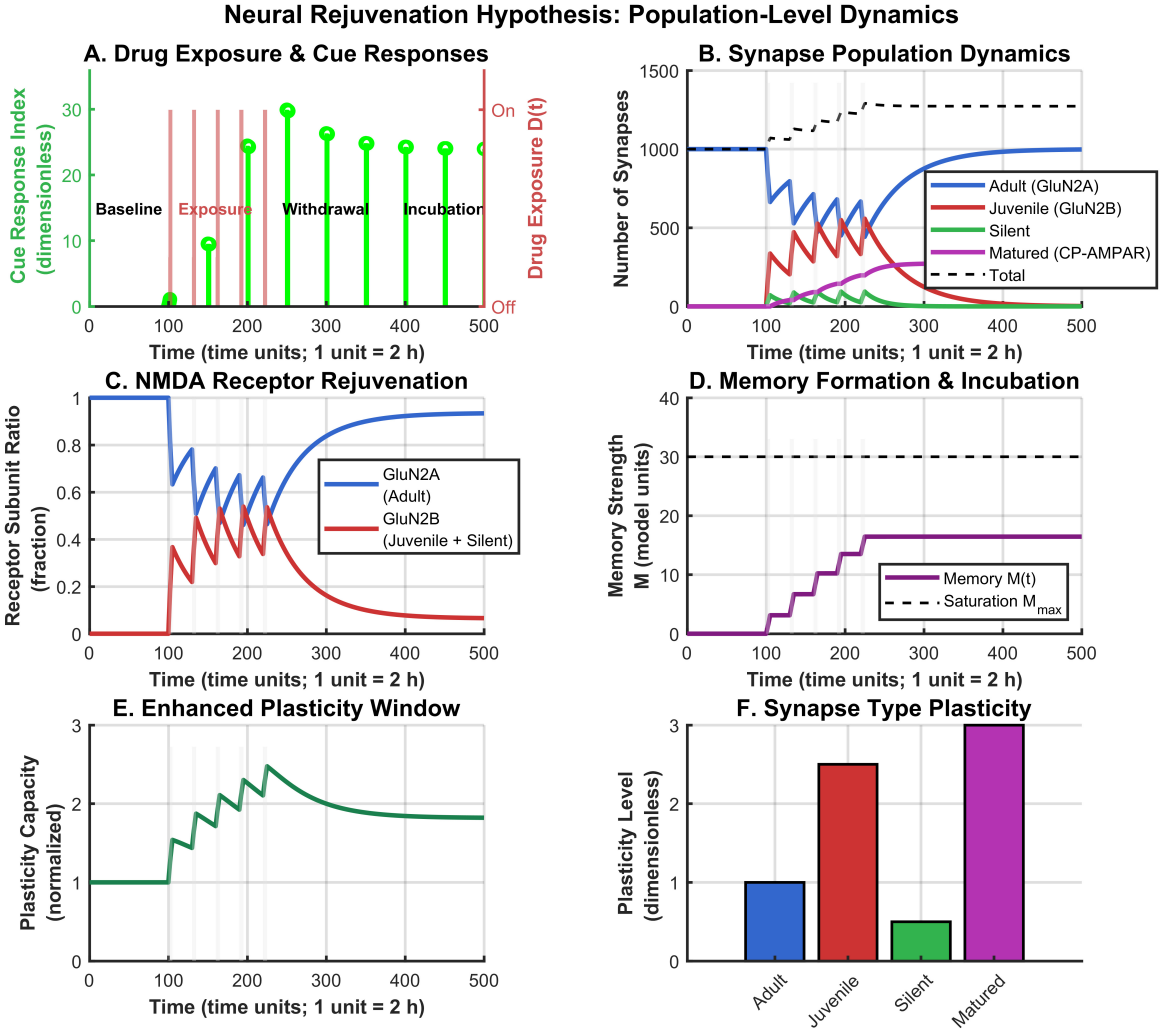
Population-level neural rejuvenation dynamics. **(A)** Drug exposure protocol with cue responses. **(B)** Synaptic population dynamics showing dynamic total (dashed line). **(C)** NMDA receptor composition shift. **(D)** Memory formation with saturation line. **(E)** Plasticity capacity. **(F)** Synapse type potentiation efficacy (weights reflect LTP amplification, not ease of modification).

The computational framework demonstrates coordinated reorganization of synaptic populations that qualitatively parallels experimental observations of neural rejuvenation (Figures 2A–F). At baseline, the entire theoretical population consisted of adult-type synapses (*N*_0_ = 1, 000 synapses). The simulated drug exposure protocol involved five intermittent exposures between time units 100–220, followed by extended withdrawal until time unit 500.

Upon simulated drug exposure initiation, rapid conversion to juvenile-like states occurred in the model, with the juvenile population increasing substantially during exposure periods, reaching peak levels of approximately 500 synapses. This Adult → Juvenile conversion reflects cocaine-induced GluN2A-to-GluN2B receptor subunit switching. Concurrent with this conversion, *de novo* silent synapse generation emerged during exposure periods, reaching maximum populations of approximately 400 synapses (constrained by the carrying capacity *K*_max_ = 500).

**Figure 3:**
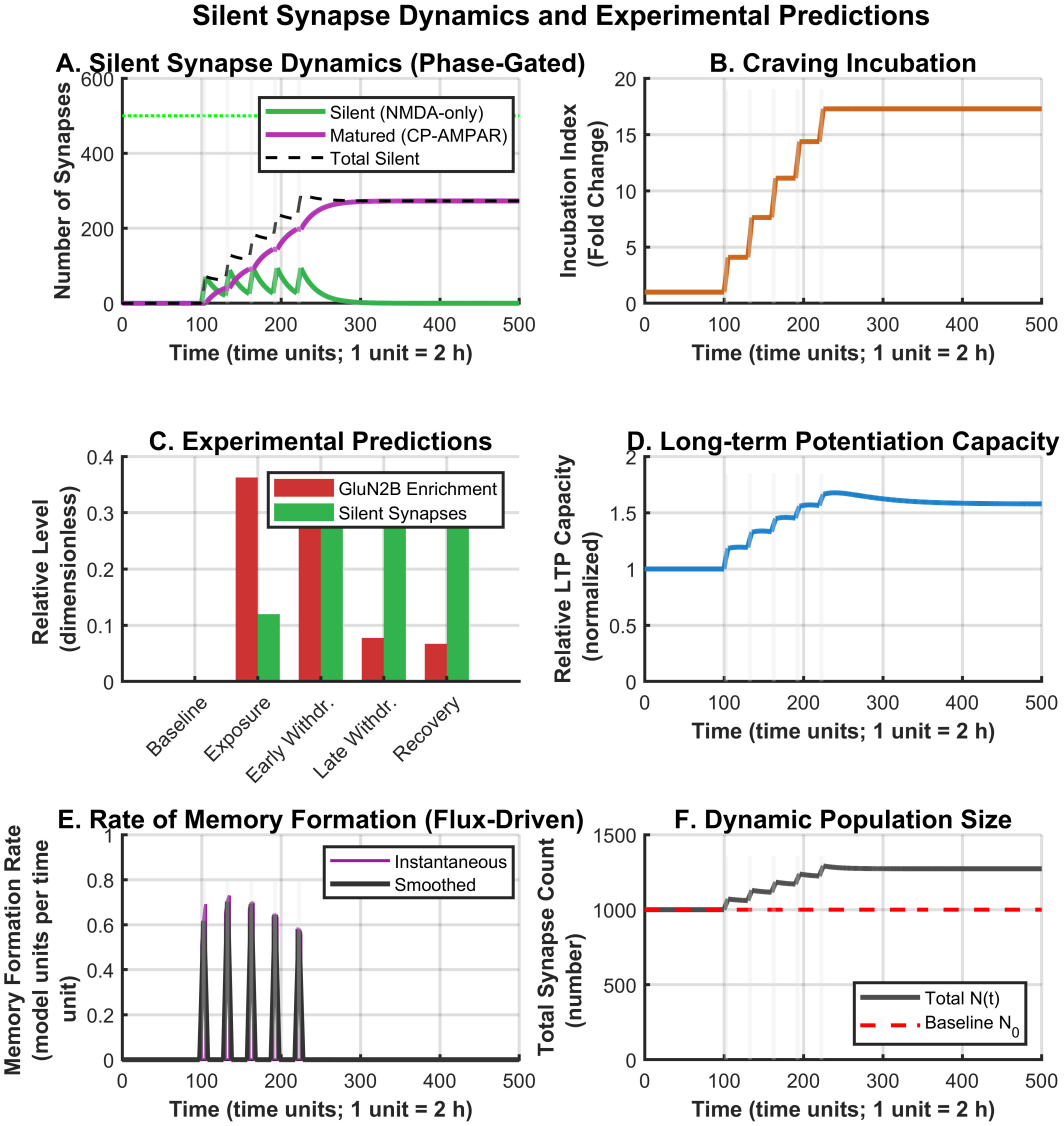
Silent synapse dynamics and experimental predictions. **(A)** Phase-gated silent synapse dynamics. **(B)** Craving incubation index. **(C)** Experimental predictions (end-of-phase snapshot values). **(D)** LTP capacity. **(E)** Flux-driven memory formation rate. **(F)** Dynamic population size.

Figures 3A–F summarizes the model's silent synapse dynamics, incubation-related measures, experimental predictions, and overall synapse population changes across phases. Importantly, the **total synapse population increased dynamically** from the baseline of 1,000 to approximately 1,400 synapses during the exposure phase (Figure 3F), reflecting *de novo* synaptogenesis. This 40% increase is consistent with experimental observations (Huang et al., 2009). During withdrawal, the total decreased to approximately 1,300 synapses due to pruning of unmaturated silent synapses, but remained elevated above baseline due to the persistent population of matured synapses.

During withdrawal, the juvenile population gradually declined (Juvenile → Adult recovery via homeostatic mechanisms) while a significant population of matured silent synapses emerged and stabilized at approximately 300 synapses by simulation end. Critically, due to the phase-gated equations, maturation and pruning occurred *only* during withdrawal periods, consistent with experimental observations (Lee et al., 2013).

### 3.2 NMDA receptor composition shifts reflect developmental rejuvenation

The simulated synaptic population transformation corresponded to profound changes in theoretical NMDA receptor subunit composition (Figure 2C). Under baseline conditions, the model exhibited mature characteristics with GluN2A receptors comprising approximately 80% of the population and GluN2B receptors representing approximately 20%, consistent with adult synaptic states.

Simulated drug exposure triggered dramatic reversal of this pattern. GluN2B-containing receptors increased rapidly during exposure periods, reaching peak levels of approximately 90% (reflecting contributions from juvenile synapses, GluN2B-rich silent synapses, and early mature synapses that retain some GluN2B), while GluN2A receptors declined correspondingly. During withdrawal, gradual recovery toward adult-like ratios occurred, though GluN2B levels remained elevated at approximately 40% compared to baseline by simulation end.

### 3.3 Progressive memory strengthening through biphasic mechanisms

The model's memory formation index demonstrated continuous enhancement throughout exposure and withdrawal phases via distinct theoretical mechanisms (Figures 2D, 3B). During simulated drug exposure, memory strength increased in discrete increments corresponding to individual exposure episodes, driven by the enhanced plasticity capacity of the rejuvenated circuit. Memory strength increased from baseline (0) to approximately 10 units by the end of the exposure phase.

During withdrawal periods, memory strength exhibited sustained growth despite absence of drug exposure, driven by the ongoing silent synapse maturation flux (Figure 3E). The memory formation rate during withdrawal depends on *k*_maturation_·*N*_silent_—the rate at which silent synapses are actively recruiting CP-AMPARs. As the silent synapse pool is depleted (through maturation and pruning), this flux decreases, naturally producing the characteristic “deceleration” of incubation observed experimentally (Pickens et al., 2011). The incubation index demonstrated approximately 18-fold enhancement relative to baseline, eventually approaching the saturation level *M*_max_.

### 3.4 Enhanced plasticity capacity persists throughout rejuvenation

Population-level changes translated into significant alterations in the model's overall plasticity capacity index (Figure 2E). Baseline plasticity was normalized to 1.0. Simulated drug exposure rapidly increased total plasticity capacity to peak levels of approximately 2.0–2.5 during active exposure periods, reflecting the enhanced potentiation efficacy of juvenile synapses (GluN2B-mediated Ca^2+^ dynamics) and accumulating mature synapses (CP-AMPAR high conductance).

During withdrawal, plasticity capacity remained elevated at approximately 1.8–2.0 above baseline due to persistent populations of matured silent synapses. This sustained elevation illustrates how rejuvenation effects might theoretically persist long after drug exposure cessation, creating a prolonged “vulnerability window” for relapse. This is also reflected in the increased LTP capacity (Figure 3D).

### 3.5 Silent synapse dynamics drive theoretical incubation mechanisms

Silent synapse populations underwent distinct temporal evolution that provides mechanistic insight into incubation phenomena (Grimm et al., 2001; Kalivas and O'Brien, 2008) (Figure 3A). NMDA-only silent synapses were rapidly generated *de novo* during simulated drug exposure, reaching peak levels of approximately 400 synapses. During withdrawal, this population declined through competitive processes of maturatio and elimination—processes that are explicitly phase-gated to occur only during withdrawal.

Matured silent synapses emerged specifically during withdrawal, representing successful CP-AMPAR recruitment. This population increased progressively, stabilizing at approximately 300 synapses by simulation end. The *rate* of this maturation process (the flux) drives memory strengthening during withdrawal, explaining why craving can continue to increase even as the rate of new mature synapse accumulation slows.

### 3.6 Parameter sensitivity analysis confirms model robustness

**Figure 4:**
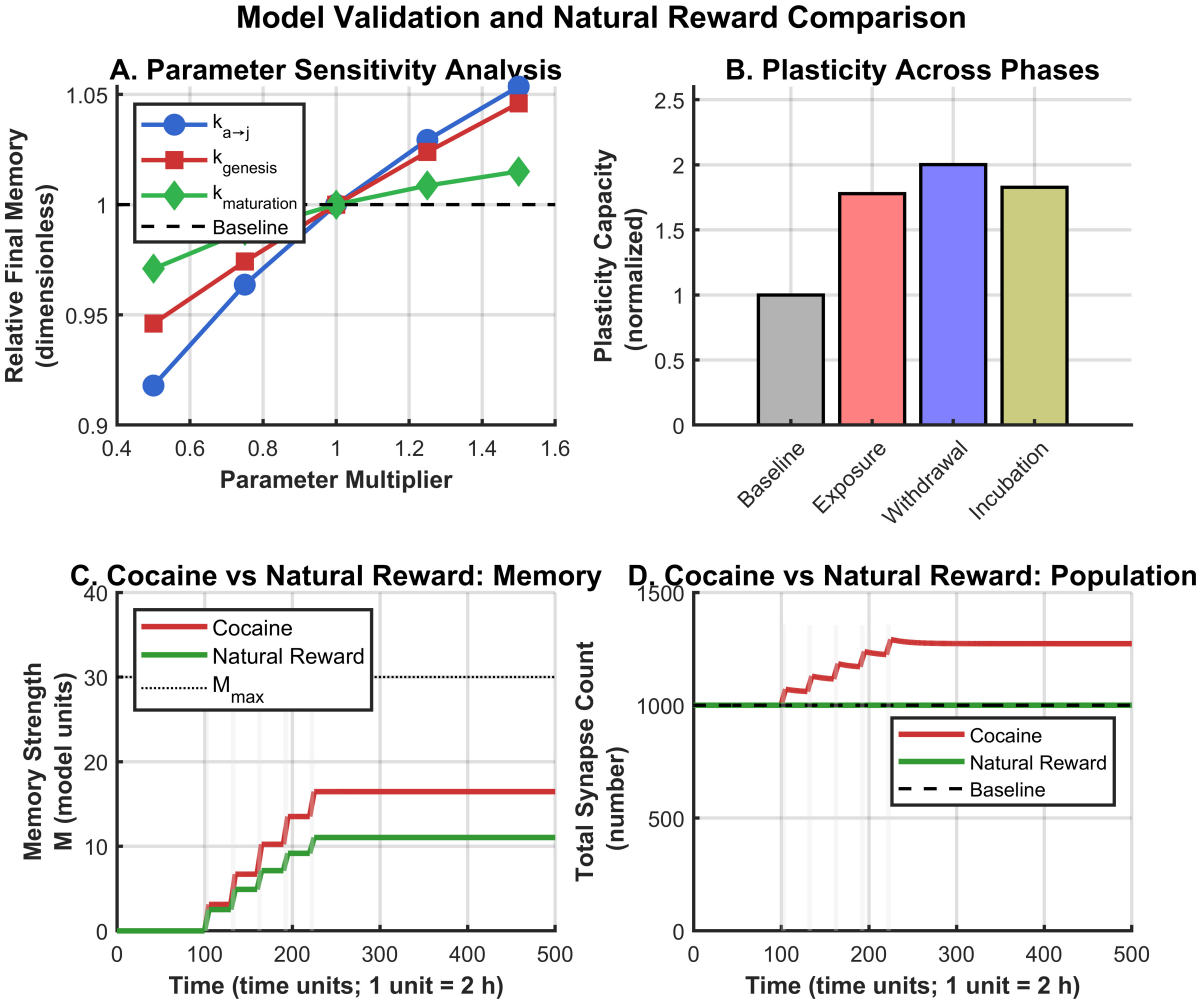
Model validation. **(A)** Parameter sensitivity analysis (±50%). Model is most sensitive to *k*_a → j_ and *k*_genesis_; insensitivity to *k*_maturation_ reflects substrate-limited (not rate-limited) memory formation. **(B)** Plasticity across phases. **(C, D)** Cocaine vs. natural reward comparison.

Systematic parameter sensitivity analysis demonstrated that the model's qualitative predictions are robust to parameter variations (Figure 4A). Varying each key parameter by ±50% produced the following findings:

- The qualitative patterns (coordinated population transitions, biphasic memory strengthening, incubation dynamics) were preserved across all tested parameter combinations.
- Quantitative outputs scaled monotonically with parameter changes, indicating smooth, predictable behavior; we did not observe qualitative regime changes (e.g., loss of coordination or incubation dynamics) within the ±50% parameter range.
- The model was most sensitive to *k*_a → j_ (rejuvenation rate), which directly determines the extent of receptor composition switching.
- The model showed moderate sensitivity to *k*_genesis_, which determines the silent synapse substrate available for maturation.
- The model was relatively insensitive to *k*_maturation_. This is because final memory strength is *substrate-limited* (determined by how many silent synapses are generated) rather than *rate-limited* (determined by how fast they mature). Even with slower maturation, the same total number of synapses eventually mature; only the timing changes.

**Initial condition robustness:** We additionally tested alternative initial states with ±10–20% juvenile synapses present at baseline (instead of 100% adult synapses). All qualitative conclusions remained unchanged, confirming that model dynamics are driven primarily by the drug exposure protocol rather than initial state perturbations.

These results demonstrate that the deterministic model produces stable, reproducible conclusions across a wide range of biologically plausible parameter values and initial conditions.

### 3.7 Natural reward comparison demonstrates drug specificity

Comparative simulations between cocaine and natural reward exposure revealed striking differences in neural rejuvenation dynamics (Figures 4C, D). Cocaine exposure produced:

- 38% increase in total synapse population due to *de novo* silent synapse generation.
- ~3-fold stronger memory enhancement compared to natural rewards.
- Pronounced incubation effect during withdrawal.

In contrast, natural reward exposure (modeled with *k*_genesis_ = 0) showed:

- No change in total synapse population (baseline maintained at 1,000).
- Substantially reduced memory enhancement.
- Attenuated incubation effect during withdrawal.

These predictions are consistent with experimental observations that natural rewards do not recruit the silent-synapse/NMDAR rejuvenation signature seen with cocaine (Huang et al., 2009).

### 3.8 Experimental predictions across addiction phases

The model generates phase-specific experimental predictions (Figure 3C) that can guide future investigations. The bar chart shows end-of-phase snapshot values for GluN2B enrichment and total silent synapse populations:

- **Baseline:** Low GluN2B (~20%), no silent synapses.
- **Exposure:** Peak GluN2B (~35%), elevated silent synapses (~12% of total).
- **Early withdrawal:** Declining GluN2B, peak total silent+mature population (~30%).
- **Late withdrawal:** Further GluN2B decline, silent pool depleted, stable mature population.
- **Recovery:** Partial normalization of GluN2B, persistent mature synapse population.

## 4 Discussion

### 4.1 Extending neural rejuvenation theory to population-level dynamics

Building on Nestler's foundational neural rejuvenation hypothesis (Dong and Nestler, 2014; Nestler, 2001), our computational approach addresses a key gap in current understanding: the population-level coordination of rejuvenation processes and their collective impact on circuit function.

Our model directly implements Nestler's core mechanisms within a population dynamics framework, with critical distinctions:

1. **Two separate processes:** Rejuvenation (receptor switching in existing synapses) versus synaptogenesis (*de novo* silent synapse generation).
2. **Dynamic total population:** The total synapse count is not fixed but increases during exposure and decreases during withdrawal.
3. **Explicit phase-gating:** Maturation and pruning occur only during withdrawal, ensuring mathematical consistency with biological observations.

### 4.2 Flux-driven incubation: a mechanistic insight

A key contribution of our model is the mechanistic explanation for continued memory/craving growth during withdrawal. The memory equation during withdrawal depends on the *maturation flux* (*k*_maturation_·*N*_silent_) rather than the absolute count of mature synapses. This has important implications:

- Memory strengthening is driven by the *active process* of CP-AMPAR recruitment, not merely the *presence* of mature synapses.
- As the silent synapse pool is depleted, the flux decreases, naturally producing incubation “deceleration.”
- This explains why blocking maturation during early withdrawal might prevent incubation more effectively than targeting mature synapses later.

### 4.3 Model predictions and experimental validation

The computational framework generates several testable predictions:

1. **Dynamic population size:** Total synapse density should increase 30%–40% during cocaine exposure and partially decrease during withdrawal.
2. **Phase-specific maturation:** CP-AMPAR recruitment into silent synapses should occur predominantly during withdrawal, not during active drug exposure.
3. **Flux-dependent incubation:** Interventions blocking maturation during early withdrawal (high flux) should be more effective than later interventions.
4. **Natural reward specificity:** Natural rewards should not produce significant silent synapse generation or population expansion.

### 4.4 Therapeutic implications

The model identifies distinct intervention windows (Figure 4B):

- **During exposure:** Prevent rejuvenation (GluN2B upregulation) or genesis.
- **Early withdrawal:** Block maturation flux (highest impact on incubation).
- **Late withdrawal:** Target existing CP-AMPAR synapses or leverage enhanced plasticity for extinction training.

### 4.5 Limitations

The model employs deterministic dynamics, simplified discrete states, and heuristic plasticity weights. The parameter sensitivity analysis demonstrates robustness across ±50% variations, and alternative initial conditions (±10%–20% juvenile at baseline) did not alter qualitative conclusions. Future refinements should incorporate stochastic dynamics, spatial organization, and systematic parameter estimation from experimental data.

## 5 Conclusions

Our computational framework demonstrates how neural rejuvenation could theoretically operate as a coordinated population process. Key features include: (1) dynamic total synapse population reflecting *de novo* synaptogenesis and pruning; (2) explicit phase-gating ensuring biological consistency; (3) flux-driven incubation mechanism explaining continued memory growth during withdrawal; and (4) natural reward comparison demonstrating drug specificity. The model produces robust qualitative predictions across parameter variations and alternative initial conditions. The model generates testable hypotheses and identifies potential therapeutic windows for intervention.

## Data Availability

The data presented in the study are deposited in the Zenodo repository, accession number: https://doi.org/10.5281/zenodo.18489543.
